# Release from cell cycle arrest with Cdk4/6 inhibitors generates highly synchronized cell cycle progression in human cell culture

**DOI:** 10.1098/rsob.200200

**Published:** 2020-10-14

**Authors:** Eleanor Wendy Trotter, Iain Michael Hagan

**Affiliations:** Cell Division Group, CRUK Manchester Institute, The University of Manchester, Alderley Park SK10 4TG, UK

**Keywords:** restriction point, cell cycle synchronization, CDK4, palbociclib, thymidine block, cell cycle

## Abstract

Each approach used to synchronize cell cycle progression of human cell lines presents a unique set of challenges. Induction synchrony with agents that transiently block progression through key cell cycle stages are popular, but change stoichiometries of cell cycle regulators, invoke compensatory changes in growth rate and, for DNA replication inhibitors, damage DNA. The production, replacement or manipulation of a target molecule must be exceptionally rapid if the interpretation of phenotypes in the cycle under study is to remain independent of impacts upon progression through the preceding cycle. We show how these challenges are avoided by exploiting the ability of the Cdk4/6 inhibitors, palbociclib, ribociclib and abemaciclib to arrest cell cycle progression at the natural control point for cell cycle commitment: the restriction point. After previous work found no change in the coupling of growth and division during recovery from CDK4/6 inhibition, we find high degrees of synchrony in cell cycle progression. Although we validate CDK4/6 induction synchronization with hTERT-RPE-1, A549, THP1 and H1299, it is effective in other lines and avoids the DNA damage that accompanies synchronization by thymidine block/release. Competence to return to cycle after 72 h arrest enables out of cycle target induction/manipulation, without impacting upon preceding cycles.

## 1. Background

Synchronized progression through the cell division cycle throughout a population supports the ability to extrapolate the biochemical and functional attributes of the synchronized bulk population back to infer behaviour in an individual cell [1,2]. Many approaches are popular. Bulk levels of DNA or cell cycle markers support fractionation of live, or fixed, cell populations into pools enriched for discrete cell cycle stages [3,4]. Although yields are low, selection synchronization based upon size, or mitotic shake off, are highly effective approaches to isolate cells in one cycle phase from a large population of asynchronous cells with minimal impact upon the proteome [5–9]. However, the ease of induction synchrony makes it the most widely applied approach.

Induction synchrony exploits the ability of transient exposure to a particular context to accumulate cells at a discrete cell cycle stage, before removal of the context simultaneously releases all cells in the population, to progress synchronously through subsequent phases of the cell division cycle [1]. In yeasts, transient ablation of cell cycle regulators through reversible conditional mutations and the addition of mating pheromones predominate [10,11]. Although the advent of analogue-sensitive versions of cell cycle kinases has introduced analogous chemical genetic approaches into human tissue culture studies [12–15], induction synchrony via serum starvation [16], contact inhibition [17] or activation of either the DNA replication, or spindle assembly, checkpoints [7,18] remain the most widely used. However, it is important to note that each form of arrest superimposes specific changes in the transcriptome and proteome upon the core cell cycle arrest signature [19–21]. These specific routes to cell cycle exit are reflected in different routes of return to the synchronized cycles [22]. Thus, the apparent reflection of normal cell cycle progression achieved with selection synchronization is yet to be matched by current approaches to induction synchronization [4,9,21].

The discovery that transient treatment with thymidine synchronized mitotic progression [18] led to protocols that sharpened the degree of synchrony by imposing a second thymidine block before releasing cells into the cycle of study [23–26]. This ‘double thymidine block’ remains one of the most popular choices; however, the power of this approach, its reliance upon the DNA replication checkpoint to arrest S phase progression, with stalled DNA replication forks, comes at a cost. Although the cell cycle arrest is robust in many lines, the stalled forks are prone to collapse over the extended arrest and subsequent attempts at repair introduce damage and chromosomal rearrangements [27–31]. There are also reports of understandable impacts upon RNA biology and proteome during the extended S phase arrest [21,32,33]. Thus, this popular approach can be of limited utility in the study of DNA replication and some transcriptional and chromatin-associated events.

When early cell cycle events are to be analysed, induction synchrony via release from a mitotic arrest in the previous cell cycle provides an attractive alternative. However, like other forms of induction synchrony that arrest *within* the cycle, prolonged cell cycle arrest will generate an imbalance in the many regulators, whose levels fluctuate with cell cycle progression, as a consequence of stage-dependent transcription and/or destruction [21,34,35]. Consequently, the next cycle may well be altered by excessive regulatory activities, or substrates, inherited from the preceding, arrested, cycle. Incisive studies by Ginzberg *et al*. [36] revealed how counter-measures to accommodate some imbalances promote adjustments in growth rates at two points in the cycle. Furthermore, prolonged mitotic arrest can initiate the atypical mitotic exit termed mitotic slippage [35,37], trigger apoptotic pathways [38] and/or leave a memory of the mitotic arrest that modifies cell cycle progression in the next cycle and beyond [39–42].

Thus, while highly informative for some questions, data obtained through traditional induction synchrony approaches, that rely upon arrest *within* the cycle, have to be interpreted with caution. They must be consolidated with complementary data from alternative approaches to reveal the commonalities that exclude the artefacts incurred in each distinct approach to synchronization.

A further challenge in synchronizing cell cycle progression throughout a population arises when there is a need to assess the impact of protein depletion, induction or replacement. It is critical to ensure that the destruction, induction or activation of a mutant variant starts after the synchronizing procedure is complete. If not, then the phenotype can be a legacy arising from perturbation of progression through the previous cycle, rather than a direct impact upon the cycle being studied. Advances in degron and PROTAC (PRoteolysis TArgeting Chimera) technologies may overcome many of these challenges [43–45]. However, even with many induction synchronization approaches, the switch from one version of a protein to another must be exceptionally rapid and complete if perturbation of the preceding cycle is to be avoided.

Inspired by the power of pheromone induction synchronization at G1 phase of yeast cell cycles [11,46], we explored the utility of induction synchrony with CDK4/6 inhibitors palbociclib, ribociclib and abemaciclib. These inhibitors arrest cell cycle progression of mammalian tissue culture cells at the restriction point in G1 phase, prior to commitment to the cell cycle [47,48]. Synchronization by induction from the natural pause point in the cycle has a number of appealing attributes. Firstly, the cell cycle programme is yet to be set in motion. Secondly, extended arrest via Cdk4/6 inhibition does not invoke compensatory changes in cell cycle or growth controls; rather it simply adjusts cell size control [36]. Finally, palbociclib-imposed cell cycle arrest has less impact upon the transcriptome than the cell type-specific changes seen upon synchronization via contact inhibition and serum deprivation [19,20].

Cdk4 and Cdk6 kinases determine commitment to the cell cycle of many cells [48]. They partner Cyclin D and the Kip family members p21 and p27 to generate active trimeric kinase complexes that phosphorylate the C terminus of the retinoblastoma (Rb) family protein [49–54]. This mono-phosphorylation supports further phosphorylation of Rb by Cdk1/Cdk2–Cyclin E and Cdk1/Cdk2–Cyclin A complexes [13]. Hypo-phosphorylated Rb binds tightly to the transcription factors of the E2F family, to block the transcription of genes required for cell cycle commitment. Rb dilution and hyper-phosphorylation relieves this inhibition, to promote transcription of cell cycle genes, including Emi1, Cyclin E and Cyclin A [55]. Induction of these Cyclins rapidly boosts Rb phosphorylation by Cyclin E and Cyclin A Cdk complexes [56]. Emi1, Cyclin E and Cyclin A complexes then seal commitment to the cycle by inhibiting the anaphase-promoting complex/cyclosome (APC/C) activating component Cdh1, thereby stabilizing APC/C^Cdh1^ targets, including Cyclin A [57–61].

The key role played by Cdk4–Cyclin D and Cdk6–Cyclin D in presiding over cellular proliferation, and the contrast between the ability of mice to survive genetic ablation of Cdk4, Cdk6 and Cyclin D and the addiction of cancer lines to these kinases, prompted the development of clinically successful Cdk4/6 inhibitors [62–66]. These inhibitors bind to the inactive Cdk4–Cyclin D and Cdk6–Cyclin D dimers, rather than the active trimeric complexes, yet they impose a very efficient cell cycle arrest [54]. This counterintuitive impact has been proposed to stem from the sequestration of the inactive dimers, or monomeric kinases by the drugs. In this model, this sequestration releases p21 and p27 to elevate concentrations of these Cdk2 inhibitors, to a level where they block the ability of the Cdk2–Cyclin A and Cdk2–Cyclin E complexes to promote the feedback loops and S phase events that drive commitment to the cycle [48,54,67]. Each drug has a specific ‘off target’ profile. Ribociclib's impressive specificity is almost matched by palbociclib, while abemaciclib shows significant off-target impacts, with notable activities towards Cdk1, Cdk2, Cdk7 and Cdk9 complexes, that can induce an arrest in G2 alongside G1 [68]. Paradoxically, this off-target impact may account for abemaciclib's greater efficacy in some clinical settings [68,69].

We describe how Cdk4/6 induction synchrony generates highly synchronous progression through the cell cycles of a number of lines, without the marked appearance of a marker of DNA damage, foci of staining with antibodies that recognize serine 139 of the histone γ-H2AX when it is phosphorylated (γ-H2AX), that accompanies thymidine induction synchronization. The ability to return to cycle after 72 h arrest in G1 with palbociclib will support a broad range of manipulations in the arrested, non-cycling state [70]. Thus, any impacts upon progression through the cycle of study will not be a secondary consequence of perturbation of the preceding cell cycle.

## 2. Methods

### 2.1. Cell lines and cell culture

The cell lines used in this study are listed in electronic supplementary material, table S1. Upon receipt, lines were expanded and frozen into aliquots of 1 × 10^6^ cells that were expanded by at least two cycles of continual passage prior to each experiment. Unless otherwise stated, hTERT-RPE-1, A549, GP2d, LoVo, U2OS, DLD-1, HCT 116, HeLa, A172, HMBC, HEK293, HEK293T and H157 were maintained in high glucose Dulbecco's modified eagles medium (DMEM: D6546, Sigma Aldrich) supplemented with 10% FBS (Hyclone, South American Origin, SV30160.03, GE Healthcare) 2 mM GlutaMAX (Gibco, 35050061) and 1000 U ml^−1^ Penicillin/Streptomycin (Gibco, 15140122). THP1, H1299, H1975, H1437, H2052, H2452, H520, H1915, NB-19, BEAS2B and H1395 were maintained in Roswell Park Memorial Institute 1640 medium (RPMI) supplemented with 2 mM GlutaMAX (61870044, Gibco), 10% FBS (Hyclone, South American Origin, SV30160.03, GE Healthcare) and 1000 U ml^−1^ Penicillin/Streptomycin (Gibco, 15140122). MCF 10A was maintained in Dulbecco's modified eagles medium/Nutrient Mixture F-12 Ham (D6421, Sigma Aldrich), supplemented with 10% Horse Serum (16050130, ThermoFisher), 10 µg ml^−1^ Insulin (I9278, Sigma Aldrich), 0.5 µg ml^−1^ Hydrocortisone (H0888, Sigma Aldrich), 20 ng ml^–1^ human epidermal growth factor (hEGF: E9644, Sigma Aldrich) and 1000 U ml^−1^ Penicillin/Streptomycin (Gibco, 15140122). A stock solution of 1 mg ml^−1^ hydrocortisone was prepared in ethanol and a 100 µg ml^−1^ stock solution of hEGF was prepared in water. Both were stored in aliquots at −20°C. Cells were maintained at a confluency of less than 70% and populations were not passaged more than 10 times before any experiment. In figure 2*e*, where hTERT-RPE-1 cells are grown in RPMI, cells were originally grown from the frozen stock in DMEM, passaged twice in RPMI, then plated in RPMI at the start of the experiment.

### 2.2. Drug treatment

Palbociclib (PD-0332991), abemaciclib (LY2835219) and ribociclib (LEE011) were purchased from Selleck (catalogue numbers S1116, S5716 and S7440, respectively), while thymidine (T1895) and nocodazole (M1404) were from Sigma-Aldrich. Palbociclib, abemaciclib, ribociclib and nocodazole were dissolved in DMSO to generate stock solutions of 10 mM in each case. Thymidine was dissolved in water to make a stock solution of 100 mM. All stocks were stored in aliquots at −20°C.

For adherent cell lines, cells were released from the substrate by treatment with trypsin (15400054, Gibco), pelleted by centrifugation at 300*g* for 5 min before resuspension in growth media. Cells were counted using a TC20 automated cell counter (BioRad) and 2.5 × 10^5^ cells were plated in a 10 cm dish (353003, Falcon) with 10 ml media (plating density = 4.4 × 10^3^ per cm^2^). For the THP1 suspension line, cells were pelleted by centrifugation at 300*g* for 5 min, resuspended in growth media and counted, before 1 × 10^6^ cells were seeded in 10 ml media in a 10 cm dish. Cells were then incubated for 6 or 12 h (see figure legends) before drug was added.

### 2.3. Flow cytometry

For cell cycle analysis, cells were washed once with phosphate-buffered saline (PBS) before releasing from the substrate with trypsin, and washed in PBS before fixation in ice-cold 70% Ethanol and freezing at −20°C for at least 18 h up to a maximum of two weeks. Fixed cells were pelleted, washed three times in PBS at room temperature before 50 µl 100 µg ml^−1^ RNase (NB-03-0161, Generon) was added to the final pellet, followed by 500 µl 50 µg ml^−1^ propidium iodide (P4170, Sigma-Aldrich) dissolved in PBS. Samples were analysed within a window of between 30 min and 6 h after addition of propidium iodide. Data were acquired on a BD LSR II flow cytometer (BD Biosciences) using FACSDiva™ software (BD Biosciences) and analysed with FlowJo software (BD Biosciences). A total of 1 × 10^4^ cells were counted for each sample.

S phase status was monitored by measuring 5-ethynyl-2'-deoxyuridine (EdU) incorporation with the Click-iT™ Plus EdU Alexa Fluor 488 Flow Cytometry Assay Kit (ThermoFisher, C10632) according to the manufacturer's instructions. To monitor cumulative DNA content between the time of release and the point of fixation in figure 9*c*, 1 µM EdU was added at the time of release from palbociclib. Cells were trypsinized and washed with 3 ml of 1% BSA in PBS, then incubated with 100 µl of Click-iT™ fixative for 15 min, pelleted, washed with 3 ml of 1% BSA in PBS and left at 4°C overnight. Cells were resuspended in 100 µl of 1× Click-iT™ permeabilization and wash reagent and incubated with 500 µl of Click-iT™ Plus reaction cocktail for 30 min, before washing once with 3 ml of 1× Click-iT™ permeabilization and wash reagent and resuspended in 500 µl of 1× Click-iT™ permeabilization and wash reagent. FxCycle Violet stain (F10347, Invitrogen) was added to a final concentration of 1 µg ml^−1^. Samples were analysed on a BD LSR II flow cytometer (BD Biosciences) using FACSDiva software (BD Biosciences) and analysed with FlowJo software (BD Biosciences). A total of 10^4^ cells were counted per sample. For quantification, the peak of 2 N cells was used to gate an assessment of 2 N DNA content, as the overlap between S phase and G2/M made it challenging to categorically assign cell cycle status on the basis of 4 N DNA content alone. Thus, throughout the manuscript, we monitor cell cycle progression as a reduction in 2 N DNA content.

### 2.4. Immunofluorescence

Cells were grown on 13 mm coverslips (No. 1.5, VWR, 631-0150P) in 10 cm dishes (353003, Falcon) using the appropriate growth conditions. For EdU staining, the Click-iT™ EdU Cell Proliferation Kit for Imaging, Alexa Fluor™ 594 dye (Invitrogen, C10339) was used. One hour prior to harvesting cells, 10 µM EdU was added to the growth media. Cells were washed in PBS before fixing for 20 min in 2% paraformaldehyde in PBS, before three washes in PBS + 0.1% Tween and storage in PBS + 0.1% Tween at 4°C overnight. Permeabilization in PBS + 0.5% Triton-X-100 was followed by three washes in PBS + 0.1% Tween. The Click-iT™ reaction for EdU detection was performed according to the manufacturer's instructions. Cells were washed three times in 5% Bovine Serum Albumin (BSA; ThermoFisher Scientific, 11423164) in PBS then incubated with primary antibody to H2AX (Ser139), clone JBW301 (Millipore Cat no. 05-636, RRID:AB_309864) at a dilution of 1 in 500 for 1 h before three washes in PBS + 5% BSA and incubation with a 1 in 500 dilution of goat anti-mouse IgG Alexafluor 488 antibody (Thermo Fisher Scientific Cat no. A32723, RRID:AB_2633275), and 2 mg ml^−1^ 4′,6-diamidino-2-phenylindole (DAPI, ThermoFisher Scientific, 11916621) for 1 h. After a further three washes in PBS + 0.1% Tween, coverslips were mounted by inversion onto 2 µl of Vectashield Antifade Mounting Medium (Vector Labs H1000). Slides were analysed using an Axioskop2 (Zeiss, Inc.) microscope.

### 2.5. Protein analysis

To collect hTERT-RPE1 protein samples, culture medium was removed, by aspiration, from a 10 cm plate, before two washes in PBS and addition of 400 µl TruPAGE LDS sample buffer (Sigma-Aldrich, PCG-3009) containing complete protease inhibitor cocktail (Roche, 11697498001), PhoSTOP (Sigma-Aldrich, 4906837001) and DTT Sampler Reducer (Sigma-Aldrich, PGC-3005) to the plate. Cells were scraped from plate for transfer into a 1.5 ml microfuge tube and snap frozen in liquid nitrogen for storage at −80°C. For THP1, cells were centrifuged at 300*g* for 3 min and the pellet resuspended in sample buffer as above, frozen in liquid nitrogen and stored at −80°C. Samples were heated at 70°C for 10 min before loading on a 10 cm, 10% precast TruPAGE gel (Sigma-Aldrich PCG2009) with TruPAGE SDS running buffer (Sigma Aldrich PCG3001) and transferred to PVDF membrane (BioRad, 1 620 177) by wet transfer with TruPAGE transfer buffer (Sigma-Aldrich PCG3011). Membranes were blocked in 5% milk and incubated in 2% BSA with primary antibodies to GAPDH (Cell Signaling Technology Cat no. 13084, RRID:AB_2713924), Eg5 (Sigma Aldrich Cat no. SAB4501650, RRID:AB_10747045), Wee1 (Cell Signaling Technologies Cat no. 13084, RRID:AB_2713924) or pHH3 ser10 (custom antibody to peptide ARTKQTARKS*TGGKAPRKQLASK: Eurogentec) overnight at 4°C. After washing, membranes were incubated for 1 h at room temperature, with the appropriate secondary antibody (Anti-mouse IgG phosphatase-conjugated antibody (Sigma-Aldrich Cat no. A3688, RRID:AB_258106), or Anti-rabbit IgG phosphatase-conjugated antibody (Sigma-Aldrich Cat# A3687, RRID:AB_258103)), washed and developed with chromogenic 5-Bromo-4-chloro-3-indolyl phosphate (BCIP, Sigma-Aldrich, B6149).

## 3. Results

### 3.1. Efficient synchronization of hTERT-RPE1 cell cycle progression with transient palbociclib treatment

The telomerase immortalized hTERT-RPE1 cell line is widely used for cell cycle and mitotic studies, yet is refractory to double thymidine block induction synchronization. We, therefore, chose this popular line to assess the efficiency of G1 arrest and release by transient exposure to palbociclib.

**Figure 1.**
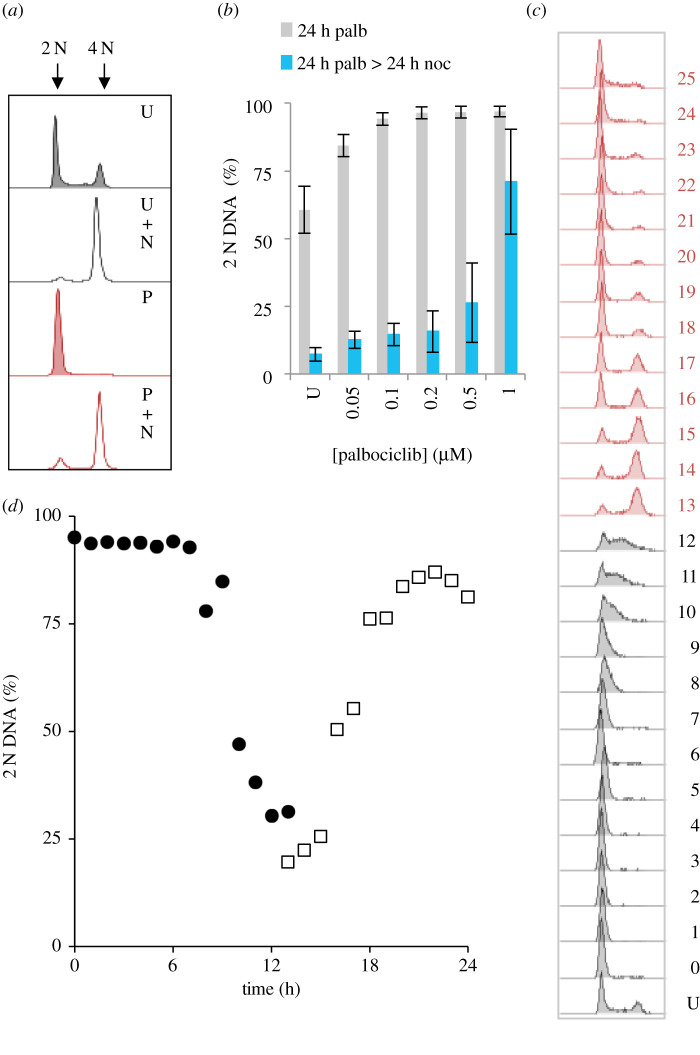
Palbociclib induction synchrony of hTERT-RPE1 cells. (*a*) hTERT-RPE1 cells were grown to 1.5 × 10^4^ cells cm^2^ in DMEM (+10% serum), trypsinized and plated into 10 cm dishes at 4.4 × 10^3^ cm^−2^. Six hours later, 150 nM palbociclib was added to the culture. After 24 h, cells were washed twice with pre-warmed medium before the addition of pre-warmed medium that contained 330 nM nocodazole before incubation for a further 24 h. Samples (one 10 cm dish per data point) were stained for propidium iodide FACS analysis at the following time points: just before palbociclib addition (U, untreated), at the switch from palcociclib to nocodazole medium (P) and 24 h after this switch to nocodazole (P + N). The strength of the nocodazole-induced spindle checkpoint arrest was revealed by the addition of nocodazole to an asynchronous population (U + N) for 24 h. The bimodal peak in the upper panel (U) shows 2 N (G1, left) DNA and 4 N (G2/M, right) DNA content of an asynchronous, untreated population. This experiment was repeated six times. (*b*) Cell populations were treated in the same way as (*a*) with the palbociclib concentration changed to the indicated value and one sample being left untreated. The average frequency of 2 N cells from at least three biological repeats is plotted for the palbociclib (grey bars) and nocodazole (blue bars) arrest points. Error bars show the limits of 1 s.d.. (*c*,*d*) hTERT-RPE1 cells were grown to around 1.5 × 10^4^ cells cm^−2^ in DMEM (+10% serum), trypsinized and plated into 10 cm dishes at 4.4 × 10^3^ cm^−2^. Twelve hours later, 150 nM palbociclib was added to the culture, before three washes in pre-warmed DMEM and sampling one dish every hour to generate the propidium iodide FACS profiles in (*c*) from which the plots of 2 N content shown in (*d*) were derived. The numbers next to the plots in *c* indicate time (hours) since release with U indicating an untreated control population. The 13–25 h plots (red (*c*): open squares (*d*)) were taken simultaneously alongside the 0–12 h population (grey (*c*): filled circles (*d*)). The synchronization shown in (*c*,*d*) has been performed six times and always reveals comparable results; however, variations in the precise synchronization profiles in each experiment (as can be seen in subsequent figures in the manuscript) mean that it is not appropriate to merge them into a single dataset.

Cells were grown to 1.5 × 10^4^ cells cm^−2^ in serum supplemented (10%) Dulbecco's modified eagles medium (DMEM), before release from the substrate with trypsin and plating at a density of 4.4 × 10^3^ cells per cm^2^. Six hours later, palbociclib was added in a range of concentrations from 50 nM to 1 µM to two identical populations. Twenty-four hours later, one population was fixed, while the palbociclib containing medium for the other was replaced with pre-warmed medium containing 330 nM nocodazole, before this sample was fixed a further 24 h later. DNA content assessment by fluorescence-activated cell sorting (FACS) analysis of propidium iodide (PI)-stained samples revealed a tight arrest at the 24 h time point with 2 N DNA content at all palbociclib concentrations of 100 nM and higher (figure 1*a,b*). Cells readily released into a 4 N arrest after being arrested in G1 for 24 h with 100 nM and 200 nM palbociclib; however, release was less efficient when the arrest was imposed by 500 nM and 1 µM palbociclib (figure 1*b*) as the 2 N DNA content remained high 24 h after nocodazole addition.

Monitoring DNA content at hourly intervals after release from 150 nM palbociclib arrest revealed a synchronous progression from G1 arrest with 2 N DNA, through S phase into G2/M phases with 4 N DNA content before a return to 2 N DNA at 20 h (figure 1*c*,*d*). As with all experiments presented herein, 24, or 48, h periods were covered by sampling parallel populations indicated by black and red lines/shading in the FACS plots and circles and squares in the graphs. For example, in the 0–13 h samples of a 24 h experiment, palbociclib was removed at the start of sampling, while the release had been done 13 h earlier for samples that were simultaneously collected for the 13–24 h samples. Consequently, many graphs we present have 13 h time points from each set.

**Figure 2.**
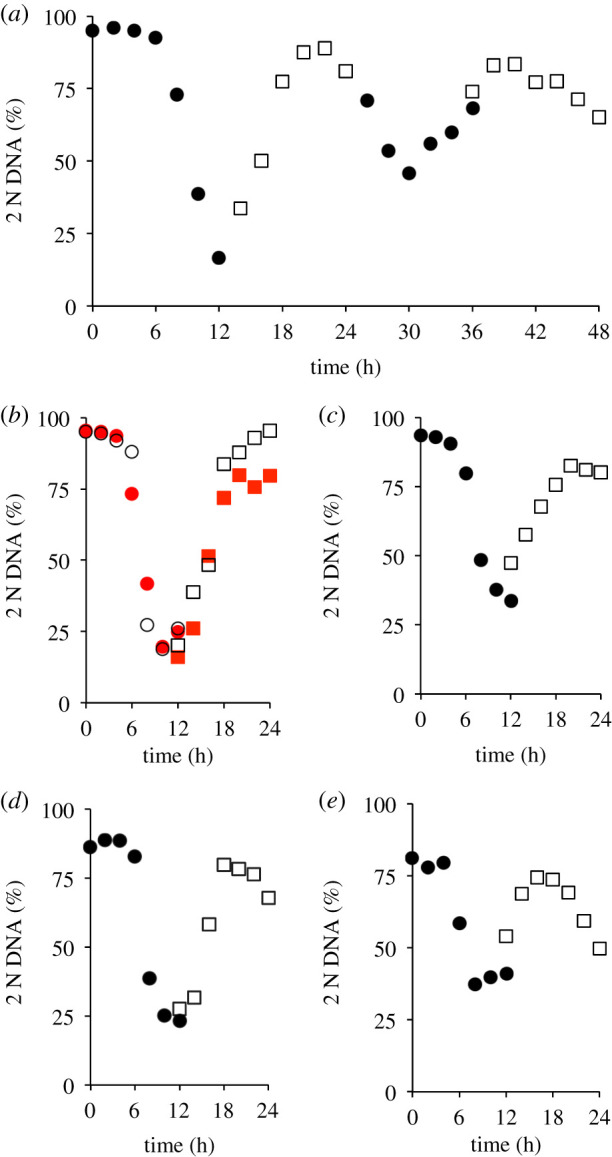
Context and perdurance for palbociclib induction synchronization. For (*a*), hTERT-RPE1 cells were grown to around 1.5 × 10^4^ cells cm^−2^ in DMEM (+10% serum), trypsinized and plated in 10 cm dishes at 4.4 × 10^3^ cm^−2^. Twelve hours later, 150 nM palbociclib was added. Twenty-four hours after palbociclib addition, cells were washed twice with medium that did not contain any palbociclib before incubation in pre-warmed DMEM (+10% serum) with sampling one dish every 2 h for propidium iodide FACS staining to generate the profiles in 12 h batches covering a 48 h release period of the same population of cells. Samples for the 0–12 (filled circles), 14–24 (open squares), 26–36 (filled circles) and 36–48 (open squares) were taken in parallel from subpopulations to which the palbociclib had been added at staggered intervals. This experiment was performed twice, with similar results in each iteration. (*b*–*e*) Plots derived from the same population of cells. For (*b*), hTERT-RPE1 cells were grown to 1.5 × 10^4^ cells cm^−2^ in DMEM (+10% serum), trypsinized and plated in 10 cm dishes at 4.4 × 10^3^ cm^−2^. Twelve hours later, 150 nM palbociclib was added. Twenty-four hours after drug addition, the cells were washed twice with medium before incubation in pre-warmed DMEM (+10% serum) that lacked palbociclib and two batches of the same population of hTERT-RPE1 cells were followed, sampling one 10 cm dish for each data point. In one batch (open symbols), 150 nM palbociclib was re-added to the population 12 h after the initial release from palbociclib, while the other was left to transit the restriction point into the second cycle (red). For (*c*), the density at which cells from the same population used for the initial plating in (*b* and *d*) were plated at a fourfold higher density of 1.76 × 10^4^ cells cm^−2^ in 10 cm dishes. (*d*) The expansion of the same starting population used in (*b*) and (*c*) lacked one cycle of splitting so that the starting population that was synchronized in (*d*) had been grown to confluence (early contact inhibition) before plating 12 h before palbociclib addition. (*e*) Cells from the same vials used to seed the populations used in *b*–*d* were grown in RPMI for two passages, alongside the cells used in *b*–*d*, before the entire synchronization outlined in figure 1*c* was conducted in RPMI. For (*b*–*e*), samples were simultaneously taken from two batches of the same population: palbociclib was added to one at the start of a 12 h sampling period (circles), while it had been added to the other 12 h earlier (squares). For the FACS plots from which these 2 N DNA contents were derived, see electronic supplementary material, figures S1 and S2.

As the second cycle after release is less likely to be influenced by the physiological challenges of induction synchrony, it is often desirable to monitor this second cycle, rather than the first cycle after release [1]. We, therefore, extended our assessments to monitor total DNA content over 48 h after release from 24 h treatment with 150 nM palbociclib. A notable degree of synchrony persisted in the second cycle with 2 N cells declining to constitute 46% of the population 30 h after release (figure 2*a*; electronic supplementary material, figure S1). Thus, it will be possible to monitor some trends in biochemical behaviour associated with cell cycle progression in the second cycle after release.

When studying events at the end of the cycle, the fastest cycling cells in the population will progress into the next cell cycle to initiate a second round of cell cycle events before the events in the first cycle are completely finished in some members of the population. This natural and subtle variation in the timing of cell cycle progression generates a merged dataset, in which information from the second cycles of some cells is superimposed upon that from the cells that are still approaching the end of their first cycle. In this way, the finer points of the kinetics of change in the first cycle can be obscured by overlapping events in the subsequent cycle. One simple solution to overcome this confusion would be to ensure that exit from the first cycle was blocked. This can be easily achieved by re-addition of palbociclib mid-way through the first cell cycle. We, therefore, asked whether such re-addition of palbociclib, after release from the first arrest, would compromise the progression through the observed cycle. Encouragingly, a second application of Cdk4/6 inhibitor 12 h after release had no impact upon progression through the cycle under study (figure 2*b*), compare the red (no re-addition) and open black symbols (palbociclib added back at 12 h: electronic supplementary material, figure S2a,b). Thus, palbociclib re-addition can be used to insulate observations from consequences of entrance into the next cycle, thereby giving greater insight into the kinetics of cell cycle events.

### 3.2. Growth conditions are key for optimal synchronization

The impact of metabolism, growth control and quiescence upon the control of commitment to the cell cycle prompted us to assess the impact of context upon the efficiency of palbociclib induction synchronization of hTERT-RPE1 cells. There was a notable reduction in the efficiency of synchronization of hTERT-RPE1 cells when the density of the population seeded onto plastic 6 h prior to palbociclib addition was increased fourfold to 1.76 × 10^4^ cells cm^−2^. At this density, the proportion of the population with a 2 N DNA content only declined to 38% rather than the dip to 16% in the identical population plated at 4.4 × 10^3^ cm^−2^ (figure 2*c*; electronic supplementary material, figure S2c). The efficiency of both arrest and release was also compromised when the identical population used in figure 2*b*,*c* had been grown to confluence to induce ‘contact inhibition’ before splitting to generate the populations that were arrested for the synchronization (declined to only 23% 2 N, figure 2*d*; electronic supplementary material, figure S2d). Finally, in simultaneous studies of cells from the same initial population that had been passaged twice in Roswell Park Memorial Institute 1640 Medium (RPMI) before synchronization in this medium*,* fewer cells arrested cell cycle progression after 24 h in Palbociclib (81% versus 95%) and the proportion of 2 N cells only dipped to 37% of the population in RPMI, rather than the decline to 16% in DMEM (figure 2*b*,*e*; electronic supplementary material, figure S2a,e). Thus, culture conditions alter the efficiency of synchronization and, once initial studies indicate that a line is competent for synchronization, a variety of conditions should be assessed and care should be taken to ensure that cells remain within active proliferation in the expansion in the lead up to synchronization.

### 3.3. Oscillations in established cell cycle markers accompany progression through synchronized cycles

**Figure 3.**
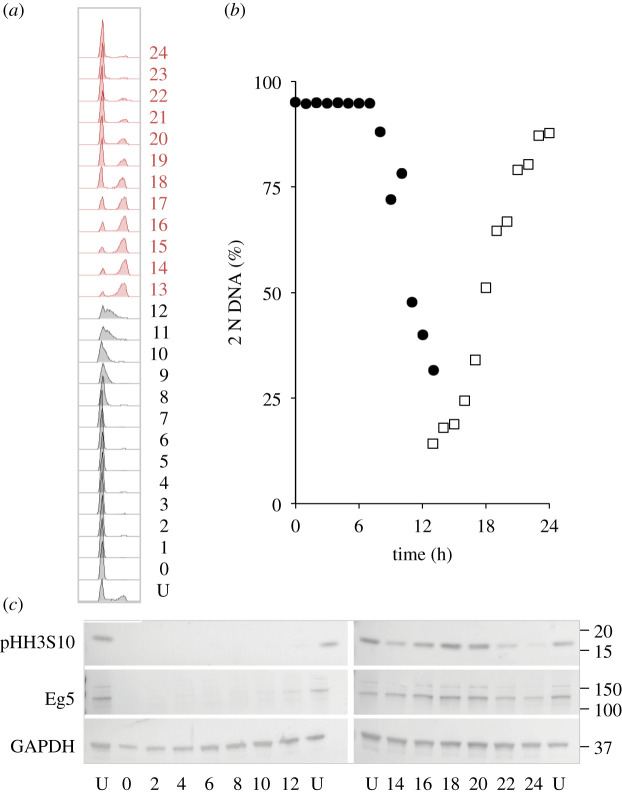
Cdk4/6 induction synchrony can reveal transient cell cycle events. hTERT-RPE1 cells were grown to around 1.5 × 10^4^ cells cm^−2^ in DMEM (+10% serum), trypsinized and plated at 4.4 × 10^3^ cm^−2^ in 10 cm dishes. Twelve hours later, 150 nM palbociclib was added. Twenty-four hours after palbociclib addition, the cells were washed twice in growth medium before the medium was replaced with pre-warmed DMEM (+10% serum) that did not contain any palbociclib. One 10 cm dish was taken for each sample every hour to generate the propidium iodide FACS profiles in 13 h batches (*a*) to gauge the fluctuations in 2 N DNA content in the population (*b*), while sampling to monitor the indicated markers by western blot every 2 h (*c*). The numbers next to the plots in (*a*) indicate hours since release with U indicating an untreated control population. Both the mitotic kinesin 5 motor protein Eg5 and phosphorylation of the serine of histone H3 at position 10 peak as cells return from the 4 N state to the 2 N state (mitosis and cell division). This plot shows one of the three repeat experiments, which revealed similar fluctuations in the same cell cycle markers.

To assess the utility of the approach for biochemical assays, extracts from a palbociclib synchronized culture were probed with antibodies to monitor markers whose levels fluctuate as cells transit cycles synchronized by other means. Anticipated fluctuations in the levels of the kinesin 5 Eg5 and phosphorylation on serine 10 of histone H3 highlight the utility of this approach to monitoring biochemical changes throughout the population (figure 3).

### 3.4. Synchronization of multiple lines with palbociclib, ribociclib and abemaciclib

**Figure 4.**
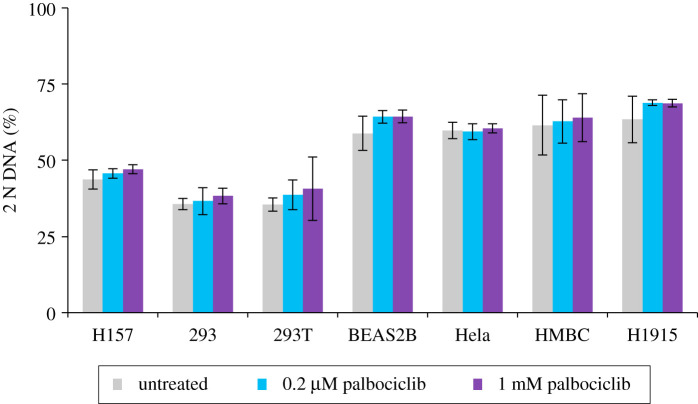
Palbociclib induction synchronization screening: refractory cell lines. The indicated cell lines were grown to around 1.5 × 10^4^ cells cm^−2^ in in the media specified in the methods, trypsinized and plated into 10 cm dishes at 4.4 × 10^3^ cm^−2^. Six hours later, the cells were treated with the indicated concentration of palbociclib or left untreated. Twenty-four hours after this, samples were stained for propidium iodide FACS analysis to gauge the proportion of the population that had a 2 N DNA content. One 10 cm dish was used for each dataset. Each plot represents the average of a minimum of four datasets (at least two biological repeats, each of which had at least two technical repeats). The error bars represent 1 × s.d.

**Figure 5.**
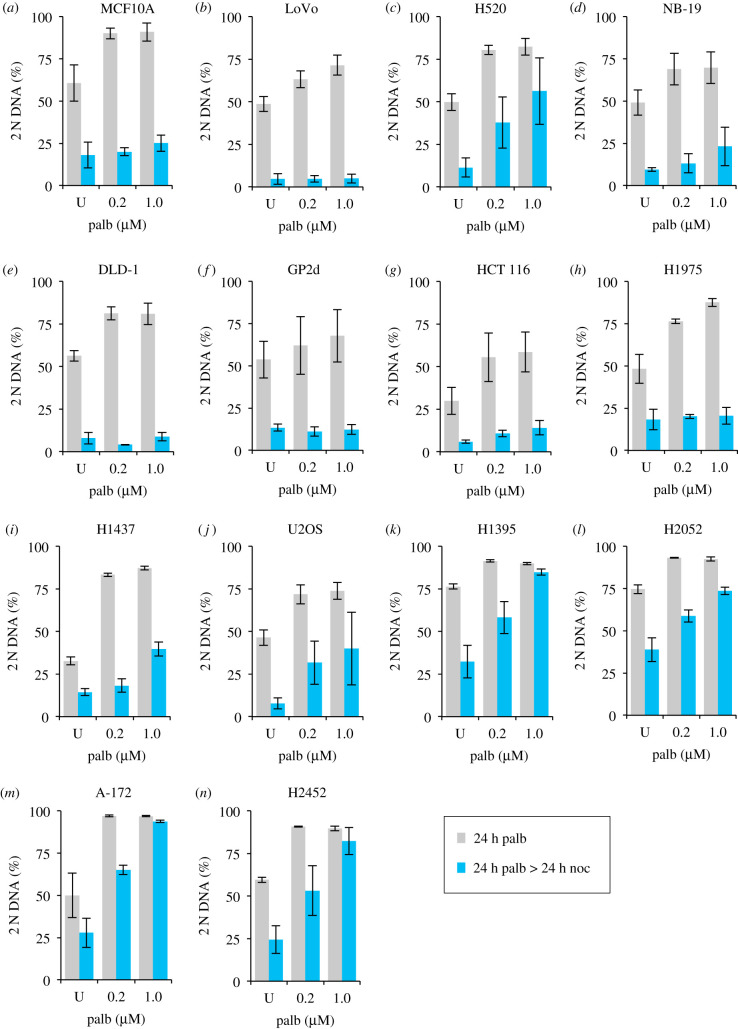
Palbociclib induction synchronization screening: responsive cell lines. Each line was grown in the media specified in the methods to around 1.5 × 10^4^ cells cm^−2^ trypsinized, and plated at 4.4 × 10^3^cm^−2^ in 10 cm dishes. Six hours later, 0.2 or 1 µM palbociclib was added to one-third of the dishes for each cell population. A control sample was left untreated. Twenty-four hours after this, cells were either fixed for staining (grey bars) or washed with fresh medium twice before incubation in 330 nM nocodazole for 24 h after which these samples were fixed and processed for propidium iodide FACs analysis alongside the samples that had been fixed 24 h earlier (blue bars). The proportion of the population that had a 2 N DNA content was calculated from the FACs profiles and plotted in the panels. The samples from the first 24 h incubation are shown in grey, while the subsequent nocodazole-treated samples are shown in blue. Each plot represents the average of at least four datasets (at least two biological repeats, each of which had at least two technical repeats). The error bars represent 1 × s.d. These experimental tests were purely exploratory in nature with the goal of finding lines for more detailed analysis in the rest of the study. Thus, these data should not be used to rule out the amenability of Cdk4/6i synchronization in the cell lines that show a partial response. We have not tested different inhibitors, media, longer incubations (in case, the cell cycles of certain lines emulate that of THP-1 of exceeding 24 h), or whether the addition of an MAP kinase inhibitor, or Cdk6 PROTAC may sharpen the responses. See Discussion for more details.

**Figure 6.**
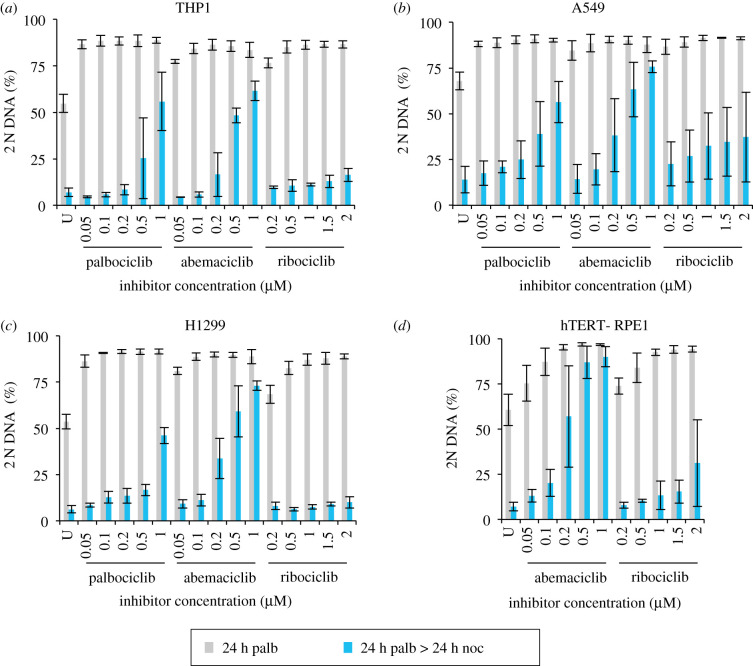
Spectrum of responses of four different lines to three Cdk4/6 inhibitors. THP1 and H1299 were grown in RPMI (+10% serum) and hTERT-RPE1 and A549 were grown in DMEM (+10% serum). Each adherent line was grown to around 1.5 × 10^4^ cells cm^−2^ before they were trypsinized and plated at 4.4 × 10^3^ cm^−2^ in 10 cm dishes. The suspension THP1 cells were grown to around 4 × 10^5^ ml^−1^, centrifuged at 300*g* for 3 min before plating at 1 × 10^5^ ml^−1^ in 10 cm dishes. One 10 cm dish was plated for each condition. Six hours later, the indicated concentration of the indicated Cdk4/6 inhibitor was added to each population. Twenty-four hours after this, samples (an entire 10 cm dish) were either fixed or washed with fresh medium twice before incubation in media containing 330 nM nocodazole for 24 h after which these samples were fixed and processed for propidium iodide FACs analysis alongside the samples that had been fixed 24 h earlier (blue bars). The proportion of the population that had a 2 N DNA content was calculated from the FACs profiles and plotted in the panels. The samples from the first 24 h incubation are shown in grey while the subsequent nocodazole arrest profiles in blue. Each plot represents the average of at least three biological repeats. The error bars represent 1 × s.d.

We next asked whether palbociclib induction synchronization would be similarly effective in other lines by assessing the efficiency of arrest and subsequent release into nocodazole of a further 24 cell lines following addition of a range of palbociclib concentrations for 24 h followed by replacement with 330 nM nocodazole medium and incubation for a further 24 h. These experiments were exploratory in nature and so were not done to the same degree of rigour as the studies of hTERT-RPE1 or the detailed A549, H1299 and THP-1 analyses described elsewhere in this manuscript. Specifically, for some lines, fewer than 10 000 cells were counted and only two biological repeats (each with two technical repeats) were conducted. These exploratory investigations revealed no appreciable impact of palbociblib upon cell cycle progression in 7 of the 24 lines (figure 4), partial responses in 9, with exploitable arrest and release profiles observed in 8: MCF10A, LoVo, H1975, NB19, DLD1 (figure 5), THP-1, H1299 and A549. Robust arrest release in the H1299, A549 and THP-1 was confirmed in more rigorous testing of at least three biological repeats, over 10 000 cell counts (figure 6).

As the different Cdk4/6 inhibitors exhibit distinct pharmacological responses [68], we compared induction synchronization profiles in four different lines to gauge the spectrum of responses and whether a reliance upon palbociclib alone as a means to judge competence for CDK4/6 inhibition induction synchrony was a valid approach, or whether other inhibitors may show even greater efficacy. A549, H1299, THP1 and hTERT-RPE1 populations were exposed to a range of palbociclib, ribociclib and abembaciclib concentrations, before the CDK4/6 drug containing medium was swapped for medium containing nocodazole 24 h into the arrest. These nocodazole-containing cohorts were then harvested 24 h after the swap into nocodazole (figure 6).

In keeping with the efficacy of palbociclib, these selected lines showed strong Cdk4/6 inhibitor induction synchrony with the two other inhibitors (figure 6). Palbociclib and ribociclib gave excellent and comparable arrest/release profiles across a broad range of concentrations. By contrast, the window of competence was much narrower for abemaciclib (figure 6). When the efficiency of G1 arrest is used to identify concentrations of drug that have comparable impact upon restriction point passage, the ability to exit the G1 arrest into the G2 block was markedly lower when the inhibition had been imposed by abemaciclib. For example, for hTERT-RPE1 cells, 200 nM abemaciclib is required to attain the same block in G1 as 100 nM palbociclib, yet there was great variation in the ability to release and an average of 57% of the population remained arrested with 2N, rather than the 15% that persists in the palbociclib-treated population (figures 1*b* and 6*d*). This inefficiency is a recurrent theme in all lines (figure 6*a–c*). Comparisons between palbociclib and ribociclib reveal subtle distinctions to suggest that, when extensive work is to be performed with a specific line, there would be merit in testing both inhibitors when fine tuning the dose–response profile.

**Figure 7.**
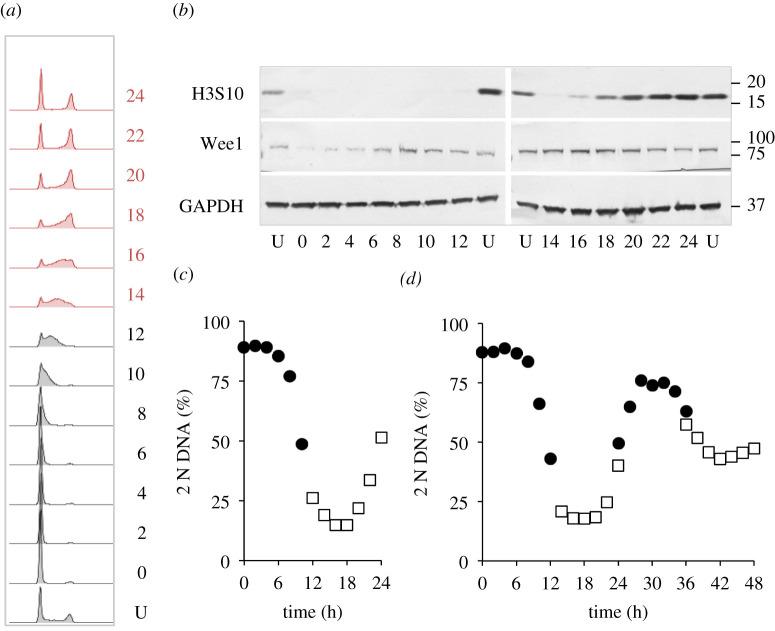
Utility of THP1 suspension cell line for cell cycle analysis. THP1 cells were grown to around 4 × 10^5^ cells ml^−1^ in RPMI (+10% serum), isolated by mild centrifugation at 300*g* for 3 min, before resuspension in RPMI at a concentration of 1 × 10^5^ cells ml^−1^ in 10 cm dishes. Twelve hours later, 150 nM palbociclib was added for 24 h before cells were isolated by centrifugation at 300*g* for 3 min and once more resuspended in RPMI. Samples (one 10 cm dish per sample) were taken every 2 h to generate the propidium iodide FACS profiles in 12 h batches (*a*) to gauge the fluctuations in 2 N DNA content in the population (*c*), while sampling to monitor the indicated markers by western blot every 2 h (*b*). The numbers next to the plots in (*a*) indicate hours since release with U indicating an untreated control population. This analysis of cell cycle samples by western blotting shown in *a*–*c* was done three times. (*d*) Cells grown as in (*a*) with the exception that sampling of 12 h batches was extended over a 48 h release period of the same population of cells. For this batch of cells, samples were only processed for PI FACs analysis of DNA content to generate the plot of the frequency of 2 N cells show in the panel. Samples for the 0–12 (filled circles), 14–24 (open squares), 24–36 (filled circles) and 36–48 (open squares) were taken in parallel from subpopulations to which the palbociclib had been added at staggered intervals. The propidium iodide FACs plots from which the data in (*d*) are derived are in electronic supplementary material, figure S3. This analysis of a 48 h progression of THP1 was done once. Note that the time taken for THP1 cells to transit the first cell cycle, under these conditions, is 32 h.

The acute myeloid leukaemia-derived line THP-1 is an attractive line for cell cycle studies because cell retrieval via pelleting avoids the challenges of harvesting from adherence to a matrix. We, therefore, assessed both the robustness of synchrony in this line (figure 7*a*,*c*, *d*; electronic supplementary material, figure S3) and the behaviour of signature cell cycle markers as a population transited a synchronized culture (figure 7*a*,*c*). As noted for hTERT-RPE1 cells in figure 3, markers oscillated with their characteristic periodicities, all be it at a slower rate, in reflection of the longer, 30 h first cell cycle of THP1 cells (figure 7*b*; compare this profile with the 22 h first cycle of hTERT-RPE1 cells, figure 2*a*).

### 3.5. Inhibitor cocktails match the efficacy of single-agent synchronization

**Figure 8.**
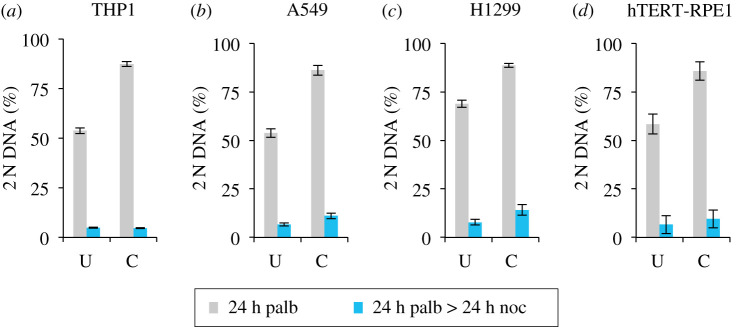
Cocktails support efficient arrest and release in four responsive lines. THP1 and H1299 were grown in RPMI (+10% serum). hTERT-RPE1 and A549 were grown in in DMEM (+10% serum). Adherent cell lines were grown to around 1.5 × 10^4^ cells cm^−2^ before they were trypsinized and plated at 4.4 × 10^3^ cm^−2^ in 10 cm dishes. The suspension line THP1 was grown to around 4 × 10^5^ ml^−1^ and centrifuged at 300*g* for 3 min before plating at 1 × 10^5^ ml^−1^ in 10 cm dishes. Four 10 cm dishes were seeded for each cell line. Six hours later, two dishes for each line were left as the untreated control, while the indicated cocktail comprising 33 nM abemaciclib + 33 nM palbociclib + 300 nM ribociclib for THP1 and H1299, 33 nM abemaciclib + 33 nM palbociclib + 67 nM ribociclib for A549 and 33 nM abemaciclib + 33 nM palbociclib + 167 nM ribociclib for hTERT-RPE1 was added to the other. Twenty-four h later, one dish for each condition was fixed for staining, while the medium in the remaining dish was replaced with medium containing 330 nM nocodazole. Media exchange for A549, H1299 and hTERT-RPE1 was achieved by aspiration while the media switch for the THP1 was achieved by mild centrifugation at 300*g* for 3 min followed by resuspension in the nocodazole-containing medium. Twenty-four hours later, these three nocodazole samples were fixed and processed for propidium iodide FACs analysis alongside the samples that had been fixed 24 h earlier. The proportion of the population that had a 2 N DNA content was calculated from the propidium iodide FACs profiles and plotted in the panels. The inhibitor cocktail arrested samples from the first 24 h incubation are shown in grey while the subsequent nocodazole arrest profiles in blue. Samples treated with the inhibitor cocktail are indicated by C, while the untreated population by U. Each plot represents the average of at least three biological repeats. The error bars represent 1 × s.d.

Off-target inhibition is a concern for interpretation of phenotypes arising from chemical perturbations. With the goal of minimizing the off-target impacts of each individual inhibitor [68], we asked whether a cocktail of all three inhibitors would be as effective as single agent? We used a mix of one-third of the most effective concentration for each individual drug for each individual drug for each cell line (as indicated in the figure legend) to test the level of cell cycle arrest and release. Encouragingly, all lines gave robust arrest and efficient release (figure 8).

### 3.6. γ-H2AX staining reveals minimal DNA damage in palbociclib induction synchrony

A major limitation of the widely used ‘double thymidine block’ approach is the accumulation of DNA damage during the early S phase arrest [28]. This damage likely arises from collapse of, and attempts to repair, the DNA replication forks that stalled because nucleotide provision was compromised [27]. The damage accrued is likely to account for the abnormal anaphase profiles in the divisions after release from thymidine block [29].

**Figure 9.**
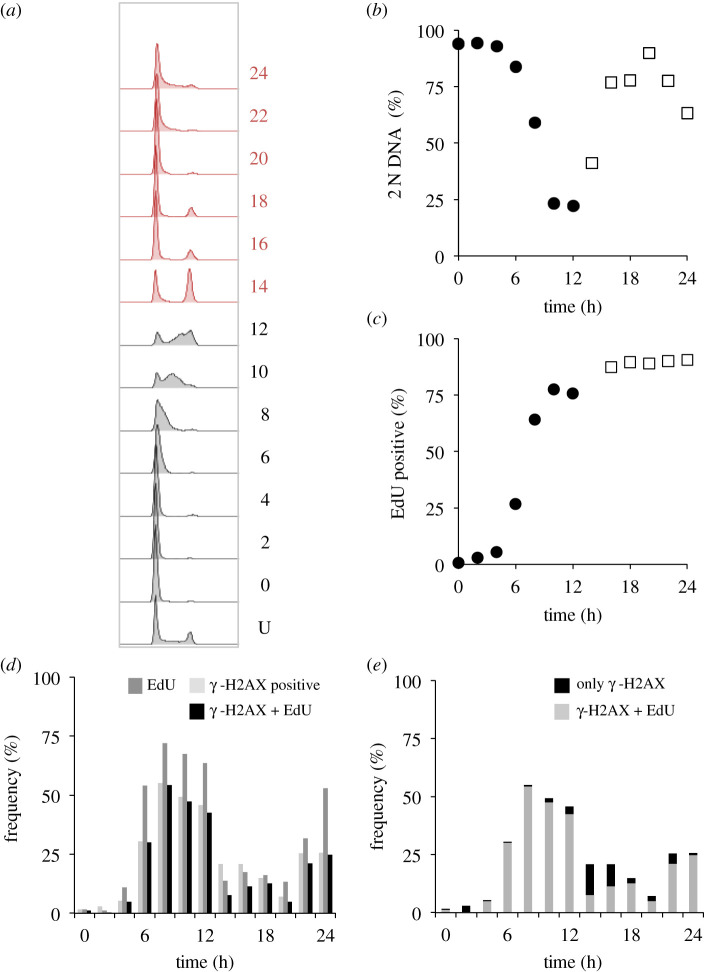
γ-H2AX foci associated with DNA replication in hTERT-RPE1 cells. hTERT-RPE1 cells were grown to around 1.5 × 10^4^ cells cm^−2^ in DMEM (+10% serum), trypsinized and plated at 4.4 × 10^3^ cm^−2^ in 10 cm dishes. Three 10 cm dishes were plated for each timepoint, one for propidium iodide staining (*a*,*b*), one for cumulatative EdU incorporation and one with coverslips for immunofluorescence. Twelve hours later, 150 nM palbociclib was added. Twenty-four hours later, cells were washed twice before incubation in pre-warmed medium that did not contain any palbociclib. At this point, one-third of dishes were left without EdU addition, 1 µM EdU was added to one-third of dishes at the time of release from palbociclib arrest, to monitor the cumulative accumulation of this marker of DNA replication (i.e. the extent of S phase progression at any one given point). In the dishes containing coverslips, 10 µM EdU was added to each dish just 1 h before these samples were fixed, in order to identify cells that were highly likely to have been actively replicating at the time of fixation. The cells with no EdU treatment were stained with propidium iodide, to monitor DNA content (*a*) that was used to calculate the value for the proportion of the population with 2 N DNA content (*b*), alongside the proportion of the population that had incorporated the EdU at any one time point (*c*). The samples from the population that had been subjected to the 1 h 10 µM EdU pulses were counterstained with γ-H2AX antibodies (*d*, *e*). The numbers next to the plots in (*a*) indicate hours since release, with U indicating an untreated control population. (*d*) Plots show the frequency of cells with staining of more than two γ-H2AX foci (light fill), EdU (intermediate fill) or those cells positive for both markers (black). (*e*) Focuses on the γ-H2AX-positive cells. The plots show the following at the indicated time points after palbociclib removal: the proportion of the whole population that stain positive for γ-H2AX alone (black, i.e. damaged cells, unlikely to be in S phase at the time of fixation), or for both γ-H2AX and EdU (grey, cells in S phase at the time of fixation). For each time point, at least 200 cells were counted to score each characteristic as a proportion of the total counted. The two populations sampled in parallel after staggered palbociclib addition are differentiated by grey and red in (*a*), closed circles and open squares in (*b*,*c*). This experiment was repeated three times with the similar outcomes each time; however, the finer kinetics of the synchrony plots differed and so it was not appropriate to combine the datasets.

We, therefore, monitored the accumulation of a marker of DNA double-strand breaks, foci of phosphorylation of *γ* -H2AX at serine 139, to assess the level of damage during palbociclib arrest and the ensuing cycle after release. As these foci form naturally during S phase, when replication forks generate double-strand breaks, we counterstained cells to identify those undergoing DNA replication in the hour before sampling by adding 10 µM of the nucleotide 5-ethynyl-2'-deoxyuridine (EdU) 1 h before processing each sample. All cells that are stained with this 10 µM EdU pulse will have been actively replicating DNA in the hour before fixation. This enabled us to distinguish EdU-positive cells with γ-H2AX foci, in which we assume the foci are a consequence of DNA replication, from those with no EdU staining, in which we assume the foci are indicative of sites of repair of DNA damage. We counted a cell as positive for γ-H2AX foci when immunofluorescence staining revealed more than two foci in a nucleus. To consolidate the insight into the timing of S phase from the assessment of total DNA content throughout the population (figure 9*a*,*b*), we monitored progression through S phase by quantifying the cumulative incorporation of EdU into the DNA after the addition of the lower concentration of 1 µM EdU at the time of release to persist throughout the experiment and label of all DNA synthesized after the release. S phase was largely complete by 16 h after release from 150 nM palbocilib (figure 9*c*).

Figure 9*d* shows plots from a population of hTERT-RPE1 cells synchronized by palbociclib arrest/release. The frequency of cells incorporating the EdU pulse (dark grey shading), those showing more than 2 γ-H2AX foci (light grey shading) and those showing staining with both (black) are indictated. For the plots in figure 9*e*, we only scored cells that contained γ-H2AX foci. The portion of cells in the population with foci, yet no EdU are shaded black. By contrast, cells S phase cells that stained positive for EdU incorporation are represented by grey shading (figure 9*e*).

These plots in figure 9*e* show that the vast majority of γ-H2AX-positive cells are those that are in the process of replicating their DNA. Very few cells in the population had the DNA damage marker, but no EdU staining. Furthermore, cells that have only just initiated S phase may not have incorporated detectable levels of the EdU nucleotide pulse, even though they will have γ-H2AX foci associated with replication forks. Consequently, the assessment by scoring a positive incorporation of the EdU pulse label will give a modest underestimate of S phase cells. Taking this into consideration, and the fact that many cells that incorporated the EdU pulse did not have any *γ* -H2AX foci (figure 9*d*), it would appear that minimal DNA damage accompanies palbociclib induction synchronization (figure 9*d*,*e*).

**Figure 10.**
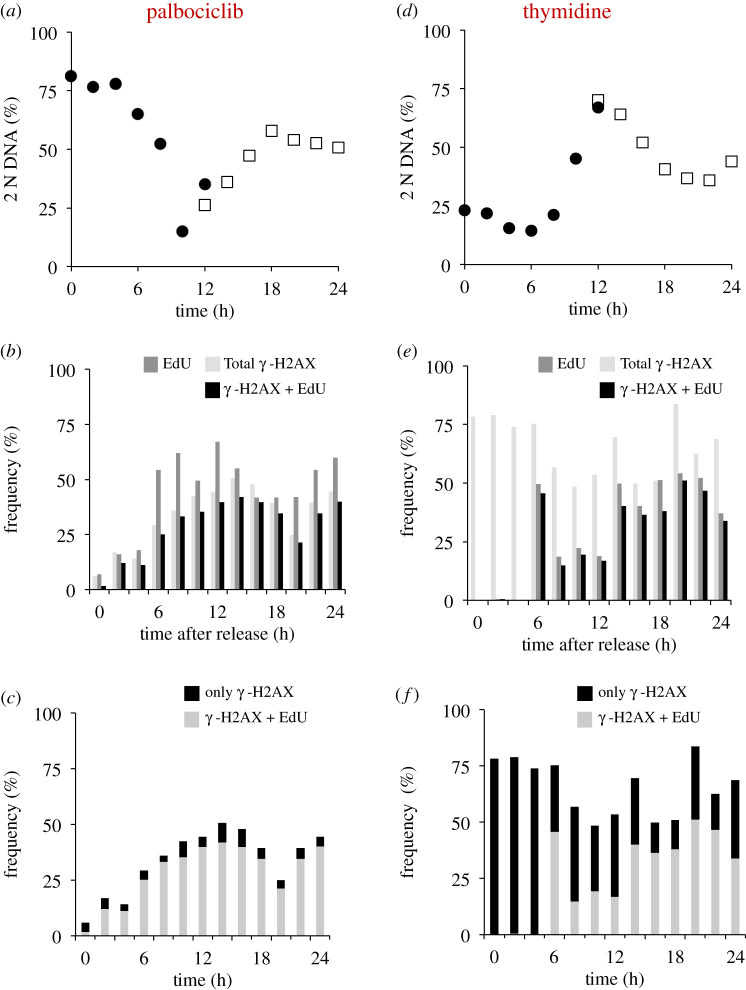
DNA damage at arrest and throughout release in thymidine but not palbociclib synchronized H1299 cells. H1299 cells were grown to 1.5 × 10^4^ cells cm^−2^ in RPMI (+10% serum), trypsinized and plated at 4.4 × 10^3^ cm^−2^ in 10 cm dishes. Twelve hours later, either 150 nM palbociclib (*a*–*c*) or 2 mM thymidine (*d*–*f*) was added, as indicated. Two 10 cm dishes were used for each condition and each timepoint, one for FACS analysis and one containing coverslips for immunofluourescence. Twenty-four hours after inhibitor addition, cells were washed twice with pre-warmed medium before the addition of pre-warmed medium that did not contain any inhibitor. Samples (the contents of one entire 10 cm dish per sample) were removed for fixation at two hourly intervals. Ten micromolar EdU was added to each dish containing coverslips 1 h before fixation, alongside processing for combined propidium iodide staining to monitor DNA content by FACs (electronic supplementary material, figure S4) from which we derived the plots of 2 N DNA content shown in (*a*). Processing the EdU staining revealed the cells that were likely to be in S phase at the time of fixation, while the γ-H2AX staining revealed cells with double-strand breaks in their nuclear DNA that could arise as a consequence of DNA damage or active replication. (*b*,*e*) Plots of the frequency of cells with staining of more than two γ-H2AX foci (light fill), EdU (intermediate fill) or those cells positive for both markers (black). (*c*,*f*) focus only on the population of γ-H2AX-positive cells. Two categories are scored: those that show only a γ-H2AX signal and no EdU signal (black) and those positive for both γ-H2AX and EdU and so are highly likely to be in S phase at the time of fixation (light). For each time point, at least 200 cells were counted to score each characteristic as a proportion of the total counted. The two populations sampled in parallel after staggered palbociclib addition are differentiated by closed circles and open squares in (*a*,*d*). This experiment was repeated three times with the similar outcomes each time; however, the finer kinetics of the synchrony plots differed and so it was not appropriate to combine the datasets.

As our unpublished experiments confirmed the widely shared view that hTERT-RPE1 cells are refractory to synchronization with thymidine (data not shown), we used H1299 to directly compare the levels of DNA damage arising during palbociclib induction synchrony and that accumulating at the cell cycle arrest and release following transient addition of 2 mM thymidine to the same starting population (figure 10; electronic supplementary material, figure S4). Although the degree of synchrony achieved by palbociclib arrest release is not as high in H1299 as in hTERT-RPE1, the results were clear.

Consistent with previous reports [28], and in stark contrast to the minimal levels of DNA damage with palbociclib induction synchronization (figure 10*a*–*c*), synchronization by exposure to a single dose of thymidine led to persistent γ-H2AX-positive scores for many cells after release from the thymidine arrest point in early S phase (figure 10*d*–*f*). Importantly, there were foci in many cells that had not incorporated the 1 h EdU pulse label at the time of fixation (the hallmark of actively replicating cells) in cells a long time after the block had been released (figure 10*e*,*f*). These data suggest that some damage that accumulated at the arrest point persisted throughout the subsequent release, beyond the period of replication. The persistence of damage is consistent with previous reports of chromosomal aberrations during divisions synchronized by thymidine induction synchrony [29].

Although we cannot exclude forms of DNA damage that do not generate γ-H2AX foci, palbociclib induction synchronization of cell cycle progression does not appear to compromise genome integrity to the same degree as thymidine based synchronization.

### 3.7. Competence to release maintained over 72 h

Removal, induction or replacement of a molecule of interest gives great insight into its function. When the manipulation is done in synchronized cultures, it is imperative that the manipulation does not occur in the preceding cycle, otherwise some of the phenotype observed could be an indirect consequence of irrelevant damage accrued in the preceding cycle as cells approach the block point. While impossible to avoid in selection synchronization, it remains a major challenge in many forms of induction synchronization that rely upon arrest *within* the cycle. One major appeal of halting cell cycle progression at a point when one cycle is complete and the next is yet to start is that the impact of any molecular manipulation of the arrested population will generate a phenotype that is a direct consequence of this manipulation upon progression through the ensuing cycle. None of the consequences will be attributable to problems in completing the cycle leading up to the block from which the cells are released.

**Figure 11.**
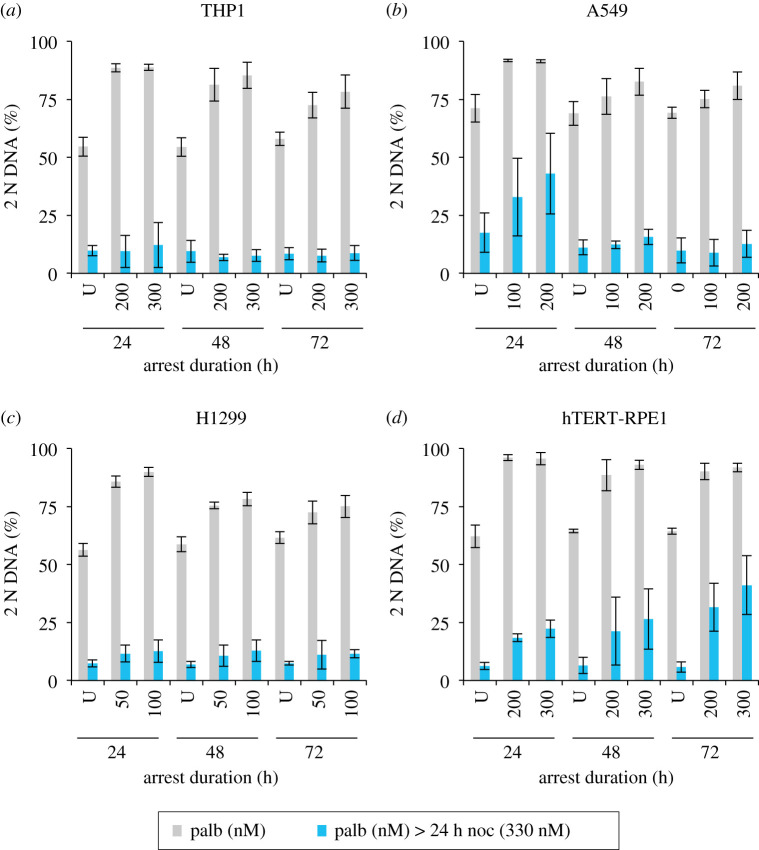
Substantial recovery from extended palbociclib-imposed cell cycle arrest. THP1 and H1299 were grown in RPMI (+10% serum), and hTERT RPE1 and A549 were grown in DMEM (+10% serum). Adherent lines were grown to around 1.5 × 10^4^ cells cm^−2^, isolated by trypsin digestion and plated at 4.4 × 10^3^ cells cm^−2^ in 10 cm dishes. The suspension cell line THP1 was grown to around 4 × 10^5^ cells ml^−1^, isolated by centrifugation at 300*g* for 3 min and plated at 1 × 10^5^ ml^−1^ in 10 cm dishes. Eighteen 10 cm dishes were plated for each cell line. Six hours later, dishes were either treated with the indicated concentration of palbociclib or left untreated. At the indicated time intervals, one plate for each condition was processed for propidium iodide FACs analysis, while another was incubated in palbociclib-free medium containing 330 nM nocodazole for a further 24 h before it too was processed for propidium iodide FACs analysis. The proportion of the population that had a 2 N DNA content was calculated from the FACs profiles and plotted in the panels. The samples after the incubation for the time shown under the plot are shown in grey, while the paired sample of cells of this population that had been released from the palbociclib arrest by medium replacement with nocodazole medium before fixation 24 h later are shown in blue. Each plot represents the average of at least three biological repeats. The error bars represent 1 × s.d.

We, therefore, compared the competence to return to cycle after Cdk4/6 inhibition for 24, 48 and 72 h. Pilot experiments established that the ability to hold the arrest, or release after arrest, varied between cell lines and so we selected different palbociclib concentrations to rigorously quantitate the competence to release following protracted arrest (figure 11). The efficiency of synchronization was assessed by the addition of the indicated concentration of palbociclib to cells 6 h after they were split from a subconfluent population and incubation for the indicated times before sampling one half of the population for FACs analysis of DNA content (grey). The palbociclib containing growth media for the other half of the population was replaced with medium containing 330 nM nocodazole before this population was sampled for FACs analysis a further 24 h later (blue). The nocodazole incubation trapped the cells released from the G1 block in the next mitosis as a consequence of activation of the spindle assembly checkpoint (SAC) [71]. While the ability to maintain the G1 arrest declined to varying degrees in the different lines, with hTERT-RPE1 being the most proficient at maintaining arrest, all lines exited the arrest to reduce the number of 2 N cells after 24 h in nocodazole (figure 11).

## 4. Discussion

Our examination of the potential of Cdk4/6 inhibition as a novel approach to induction synchrony reveals a highly effective approach to synchronization of cell cycle progression throughout a population of either adherent, or suspension, human cell lines. We believe that a number of attributes make it a valuable addition to the broad portfolio of cell cycle synchronization technologies.

The release from palbociclib-imposed cell cycle arrest at the natural decision point for cells, the restriction point, is not associated with changes in growth rates or rates of progression through the cycle into which they are released [36,72]. Rather Cdk4/6-Cyclin D activity appears to set size control [36,72]. Conceptually, this resonates with the original definition of the restriction point, as a rate-limiting gateway upon which multiple regulatory systems converge to regulate passage through the gateway into commitment to division [47].

A second appeal of this approach lies in the apparently limited impact upon genome integrity. Although the double thymidine block approach has predominated for over 50 years, the DNA damage that accompanies the arrest persists through the release (figure 10) [28,29]. This cumulative damage limits the utility of this approach for the study in several fields, including DNA replication, DNA repair and chromatin. While assessing genome integrity through the acquisition of γ-H2AX foci is not an exhaustive assessment of damage, our data suggest that Cdk4/6i induction synchrony is not accompanied by the damage that arise following release from thymidine [29]. Extensive analyses of mitotic progression following palbociclib induction synchronization revealed no sign of any of the chromosomal abnormalities that accompany thymidine induction synchronization [73] (Jon Pines and Mark Jackman 2020, personal communication). Cdk4/6i induction synchronization, therefore, has the potential to open up a number of previously intractable questions to study in synchronous cultures.

Perhaps the most important appeal of this approach is that cells can remain arrested outside of the cell cycle in full serum for very protracted timescales while retaining the competence to return to a synchronized cycle. This stasis means that a molecule of interest can be completely depleted, while cells are out of the cycle, without having any impact upon the progression through the previous cell division cycle. If a mutant version of this target was simultaneously induced, it would support highly directed questions about protein function when the cells are released to synchronously transit the ensuing cycle. The study of regulation of centriole biogenesis by Viol *et al*. [70] provides a compelling illustration of the power of protein induction in a palbociclib arrest prior to release.

While the benefits of this approach are particularly attractive, as with all approaches to cycle synchronization, disadvantages will inevitably emerge as these protocols are more widely adopted. Although it seems that cell mass accumulation at the arrest point does not impact upon cell cycle kinetics after release [36], studies in model organisms suggest that there is likely to be adaptation to modified size control at some point after commitment [74]. Recent studies suggest that adaptation is unlikely to occur after the second cycle after release [72]. The approach is also not effective in many lines when the level of control exerted by Cdk4/6 versus Cdk2 is tipped more heavily in favour of Cdk2 control [13,20,22,48,54,61–63,72,75–79].

When a particular line is refractory to Cdk4/6 induction synchronization, several approaches may switch the line to confer sensitivity to Cdk4/6 inhibition. For lines in which Cdk4/6 inhibition alone has little impact, reducing flux through to Cdk4/6 Cyclin D by reducing serum, or inhibiting the downstream MAP kinase pathways can compromise translation to reduce Cyclin D levels and tip the balance to impose cell cycle arrest by palbociclib, or Cdk2 inhibition [13,59,72,80]. The cell cycle arrest in HCT116 when Trametinib complements palbociclib is a good example of this synergy [81]. The recent revelation that the Cdk4/6 inhibitors are targeting the inactive, rather than active Cdk4 and Cdk6, complexes suggests another option for refractory lines when resistance arises from greater reliance upon Cdk6, rather than Cdk4 [54]. Cdk4/6 inhibitors impose the arrest because they sequester Cdk4 and Cdk4–CyclinD away from the Hsp90 chaperone system to reduce the number of molecules that can form an active complex with p27 [54,67,82]. The lower affinity of Cdk6 for the Hsp90 chaperone complex enables it to more readily assemble into active trimers that have no affinity for the inhibitors than Cdk4 does. This has prompted the suggestion that the predominance of Cdk6–Cyclin D-p27 trimers depletes p27 from the pools of Cdk2 to elevate Cdk2 activities and confer palbociclib resistance in cancers in which Cdk6 expression is elevated [67,83,84]. Thus, cell lines that rely upon Cdk6 rather than Cdk4 to drive passage through the gateway into the cycle will have reduced sensitivity to Cdk4/6 inhibitors. In such lines, a Cdk6-specific PROTAC may reduce reliance upon Cdk6 to impose sensitivity to Cdk4/6 inhibition and so support synchronization with the inhibitors [85]. Finally, because many cell lines will bypass the requirement for Cdk4/6 by exploiting Cdk2 to drive cells into cycle, partial inhibition of Cdk2 in these lines [13,86–88] should sensitize cells to Cdk4 inhibition. One challenge with this approach is the accompanying risk that strong Cdk2 inhibition will impact not only upon DNA replication in the preceding cycle, but upon the regulation of mitotic commitment because Cdk2–Cyclin A has been tied directly to regulation of Wee1 and the G2/M transition [13,89–91].

Given the variety of means by which resistance to Cdk4/6 inhibitors arises [48], it is perhaps easiest to empirically test whether a line will be amenable to Cdk4/6i induction synchrony. One of two simple assessments will identify a line as being Cdk4/6i induction synchrony compliant. The arrest/release into nocodazole that we show in figures 1 and 4–6 will indicate competence of most lines to synchronize by Cdk4/6i induction synchrony: a line will synchronize if a population accumulates 2 N DNA content one doubling time after inhibitor addition, before switching to 4 N DNA content a further doubling time after release into nocodazole. However, this assay relies upon the strength of the SAC [71] that can be so weak in some lines that it fails to impose a long cell cycle arrest with 4 N DNA content [92]. The second approach is independent of SAC integrity. Cells are released from palbociclib into 1 µM EdU before entrance into the next cycle is blocked by re-addition of palbociclib 12 h after release. If EdU is incorporated into most genomes throughout a population that has arrested cell cycle progression with a 2 N DNA content in response to the second dose of palbociclib, then the line will be competent for synchronization.

Our manipulations of cell density and culture history highlight the importance of growth state and culture history when customizing the synchronization protocol to gain maximum synchrony in the chosen line. The plasticity of the G1 control of commitment to the cycle is well documented. Distinct transcriptional and proteomic changes accompany cell cycle exit when a switch from the cycle is triggered by contact inhibition, serum starvation or inhibition of DNA replication, such that a cell's history alters the configuration of signalling in actively cycling cells and those returning from quiescence [19–22,59,72,80]. Thus, a recent history of contact inhibition reduces the efficiency of synchronization upon release from palbociclib (compare the profile in figure 2*b* of cells that have been kept in subconfluent culture prior to manipulation, with that if figure 2*d* which shows the same starting population of cells that had experienced a brief spell of contact inhibition). Such memory of contact inhibition is likely to have broad ranging impacts, as illustrated by the way in which it changes the loading of replication factors at origins in hTERT-RPE1 cells [93]. Consequently, care should be exercised to ensure that a population's path to synchronization has been freely dividing and as ‘healthy’ as it can be.

While we outline the utility of palbociclib induction synchrony in the study of cell cycle control and execution, pausing the cycle at the restriction point could have considerable benefit in other fields, such as synchronizing the formation of the primary cilium by serum depletion from Cdk4/6i arrest, or in studying differentiation in systems, such as haematopoesis, where a pause in G1 can be crucial for differentiation.

In conclusion, the successful quest to generate drugs to control proliferation in the clinic [65] has generated tools that will be of great utility in studies of the functional and biochemical changes that accompany and drive cell cycle progression.

## 5. Conclusion

Cdk4/6 induction synchrony is a simple, reproducible approach that will support biochemical interrogation, induction, depletion and replacement of molecules in any scale of cell line culture. Simple assays can determine competence of this approach to synchronize cell cycle progression in a novel line. We anticipate that a number of approaches may convert a recalcitrant line into a Cdk4/6i synchronization compliant line.

## Supplementary Material

Supplementary Figures

## Supplementary Material

Supplementary Table 1

## Data accessibility

This article has no additional data.
