# Supplementary material for: Release from cell cycle arrest with Cdk4/6 inhibitors generates highly synchronized cell cycle progression in human cell culture
Source: Open Biol. 2020 Oct 14;10(10):200200. doi: 10.1098/rsob.200200 (PMC7653349; doi:10.1098/rsob.200200)
Supplement: Supplementary Figures [file rsob200200supp1.pdf]

Trotter and Hagan Release from cell cycle arrest with Cdk4/6 inhibitors generates highly synchronised cell cycle progression in human cell culture
Open Biology 10.1098/rsob.20160200

Supplementary Figure 1

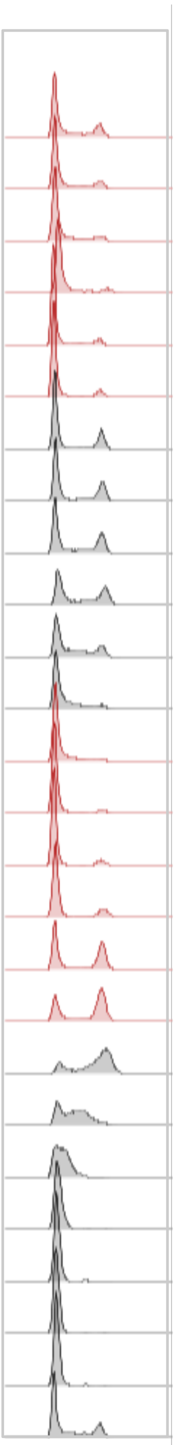

Propidium Iodide FACS profiles of synchronised populations in Figure 2a
The plots of propidium iodide FACS analysis of the populations shown in Figure 2a. The parallel samples indicated by alternating filled circles and open squares in Figure 2a are distinguished by alternating grey and red shading, respectively. Numbers indicate hours since release while U = untreated.

Supplementary Figure2

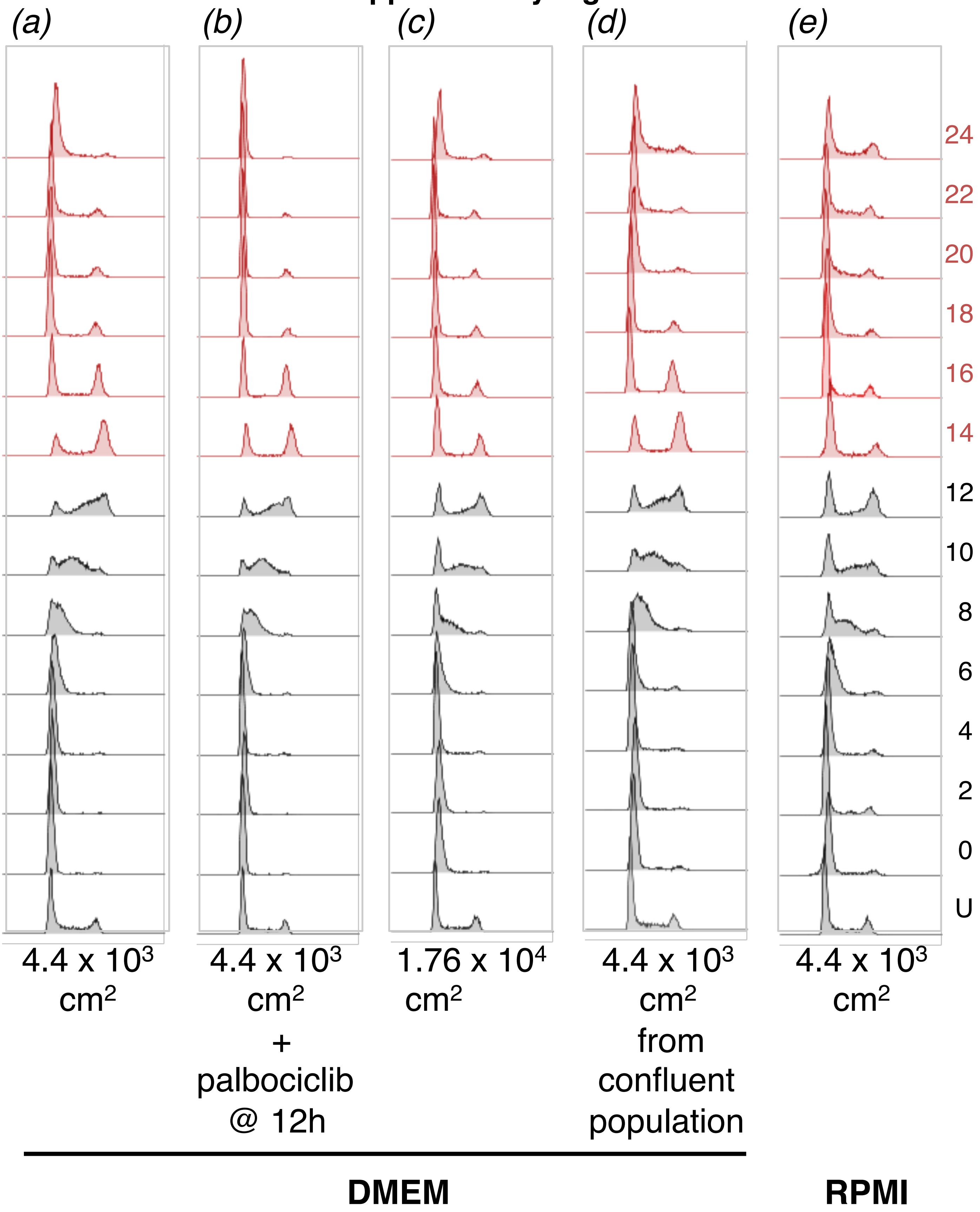

Propidium Iodide FACS profiles of synchronised populations in Figure 2b-e
The plots of propidium iodide FACS analysis of the populations shown in the respective panels of Figure 2b-e. The parallel samples indicated by circles and squares in Figure 2 are distinguished by the grey and red shading, respectively. Numbers indicate hours since release while U = untreated.

Trotter and Hagan Release from cell cycle arrest with Cdk4/6 inhibitors generates highly synchronised cell cycle progression in human cell culture
Open Biology 10.1098/rsob.20160200

Supplementary Figure 3

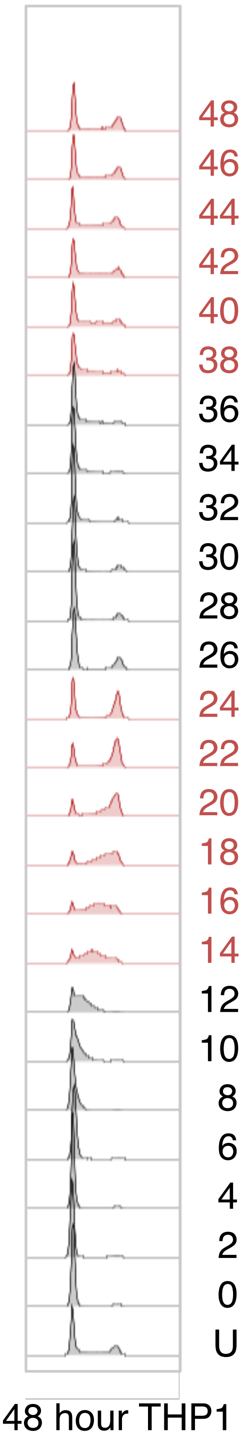

Propidium Iodide FACS profiles of synchronised populations in Figure 5d
The plots of propidium iodide FACS analysis of the populations shown in Figure 7d. The parallel samples indicated by alternating filled circles and open squares in Figure 7d are distinguished by alternating grey and red shading, respectively. Numbers indicate hours since release while U = untreated.

Trotter and Hagan Release from cell cycle arrest with Cdk4/6 inhibitors generates highly synchronised cell cycle progression in human cell culture
Open Biology 10.1098/rsob.20160200

Supplementary Figure 4

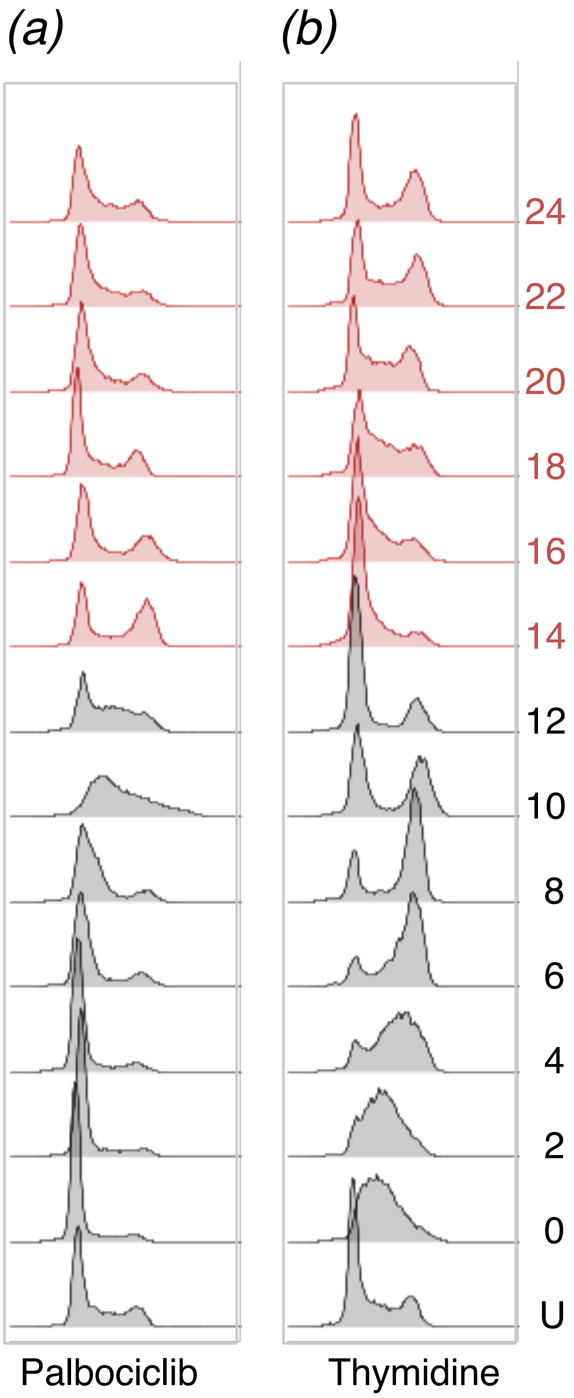

Propidium Iodide FACS profiles of synchronised populations in Figure 8
The plots of propidium iodide FACS analysis of the populations shown in the respective panels of Figure 10. The parallel samples indicated by filled circles and open squares in Figure 10 are distinguished by the grey and red shading. Numbers indicate hours since release while U = untreated.
